# The role of maturity offset in predicting aerobic and anaerobic performance outcomes in young off-road cyclists

**DOI:** 10.3389/fspor.2026.1868914

**Published:** 2026-07-22

**Authors:** Domenico Savio Salvatore Vicari, Giuseppe Scardina, Valerio Giustino, Matteo Giuriato, Antonio Sedita, Gerlando Michele Scrofani, Alessandro Mansueto, Salvador Cabeza de Vaca Gallardo, Francisco José Berral-de la Rosa, José Naranjo-Orellana, Antonino Bianco

**Affiliations:** 1 Sport and Exercise Sciences Research Unit, Department of Psychology, Educational Science and Human Movement, University of Palermo, Palermo, Italy; 2 Department of Neurosciences, Biomedicine and Movement Sciences, University of Verona, Verona, Italy; 3 Laboratory of Adapted Motor Activity (LAMA), Department of Public Health, Experimental Medicine and Forensic Science, University of Pavia, Pavia, Italy; 4 The Sport Lab, Sedita Medical Center, Caltanissetta, Italy; 5 Research and Development Technical Centre, Sicilian Regional Committee of the Italian Cycling Federation, Caltanissetta, Italy; 6 CTS-595 Research Group, Department of Informatics and Sports, Universidad Pablo de Olavide, Seville, Spain

**Keywords:** biological maturation, cycling performance, growth, peak height velocity, youth cyclists

## Abstract

**Introduction:**

Biological maturation is a crucial aspect when assessing performance in young athletes. The maturity offset, indicating the time relative to Peak High Velocity (PHV), can impact aerobic and anaerobic capacity, as well as absolute and relative power outputs. This study investigated how maturity offset affects Peak Power Output (PPO) during the power-cadence test and Maximum Power Achieved (MPA) during an incremental ramp test in young off-road cyclists.

**Methods:**

Twenty-two young off-road cyclists (15 m, 7f) were recruited. The maturity offset of each participant was calculated using the Mirwald's predictive equations for boys and girls. A bike fitting was performed on their own bikes installed on a specific bike roller to optimize joint function. Before testing, participants were asked to warm-up for 10 min at self-selected pedalling intensity and cadence. Then, they performed a power-cadence test which consisted of five maximum sprints, each lasting 6 s, using different gear ratios. Later, participants were asked to perform an incremental ramp test with an initial power output of 2.8 W*kg-1, increasing by 10 W*min-1 until exhaustion.

**Results:**

Our results showed that the maturity offset was a significant predictor of the PPO (*p* = 0.007) and of the MPA (*p* = 0.013). Sex was significantly associated with MPA (*p* = 0.004) and MPA·kg⁻^1^ (*p* = 0.002). Nevertheless, maturity offset did not appear to be a significant predictor of cyclists’ relative power-to-weight ratio.

**Conclusion:**

Maturity offset appears to be a strong predictor of absolute PPO and MPA rather than relative PPO and MPA. This suggests that maturity status mainly affects aerobic and anaerobic absolute power. However, early mature status is not necessarily associated with higher performances compared to peers with late mature status. The use of maturity offset can be an effective technique for contextualizing performance in youth, as well as for monitoring the biological developmental trajectories of young athletes.

## 1 Introduction

Biological maturation is an influencing factor of performance in adolescents, influencing both aerobic and anaerobic capacity (1). A commonly used method for assessing maturity status is the estimated age to achieve the Peak Height Velocity (PHV), obtained with the maturity offset, which represents the age from the PHV. If this value is positive, the subject has already passed the PHV; if this value is negative, the subject has yet to reach the PHV. This allows to estimate the degree of maturation in young athletes.

Maturity status can be determined directly via hand-wrist radiography or predicted via non-invasive field-based methods. Among these, the equation proposed by Mirwald et al. (2002) is the most commonly used in youth sports and is based on non-invasive anthropometric measurements such as height, weight, sitting height, and leg length, in addition to chronological age (2). Subsequently, Kozieł and Malina (2018) introduced a modified version designed to minimize the systematic error present in the original equation, particularly in subjects close to PHV (3). Although both equations offer good predictive accuracy, several researchers have noted that they can introduce bias in sports, specifically by predicting the age of PHV too early in younger subjects and too late in more developed subjects (3, 4).

The period around the PHV is characterized by morphological and functional changes. During puberty, a rapid increase in lean mass, muscle strength, and power generation occurs, along with an augment in cardiovascular capacity such as stroke volume (5). These changes are associated with an increase in absolute aerobic capacity, although, once normalized for body weight, the influence of maturation tends to decrease. At the same time, the progressive maturation of glycolytic enzyme systems and the increase in the availability of energy substrates lead to an enhancement in anaerobic capacity and lactate tolerance (6).

As for the aerobic capacity, the literature has extensively documented that VO_2_max, during adolescence, increases with age and stage of maturation. However, much of the observed variability can be explained more by changes in body composition (i.e., height, weight, and lean mass) and scale effects related to growth than by a direct maturation effect (7). Furthermore, recent studies have shown that, after correcting VO_2_max for body weight or lean mass, the impact of maturation is attenuated or even eliminated, suggesting that maturation acts primarily through anthropometric and metabolic changes (8). Similarly, anaerobic capacity is also influenced by maturation status. For example, in a study analyzing young rowing athletes, it was observed that greater maturity was associated with greater anaerobic power and reduced times in specific tests, although these relationships were largely mediated by lean mass and metabolic factors (8).

Nevertheless, the relevance of maturity offset as a predictor of aerobic and anaerobic capacity in young athletes, in particular in off-road cyclists, is still largely unexplored. In off-road cycling, aerobic and anaerobic efficiency is crucial. Indeed, the ability to generate power during intervals and to sustain prolonged loads are key factors for race performance. Previous studies in young road cyclists have shown that anthropometric and physiological variables (e.g., threshold power, VO_2_max) correlate with performance, but these studies rarely include maturity status estimation.

The aim of this study was to assess whether maturity offset could be considered as a predictor of specific aerobic and anaerobic performance outcomes in young off-road cyclists. The hypothesis was that maturity offset could be a predictor of cycling performance. In particular, subjects with a positive maturity offset could show higher performance outcomes compared to peers with negative maturity offset.

## 2 Materials and methods

### 2.1 Study design

This study is a cross-sectional design that evaluated the relationship between maturity offset and specific aerobic and anaerobic performance outcomes in young off-road cyclists.

### 2.2 Participants

Twenty-two young off-road cyclists (15 m, 7f) aged 13 to 16 years (Italian Federation categories: ES1, ES2, AL1, AL2) were recruited from the Research and Development Technical Centre of the Sicilian Regional Committee of the Italian Cycling Federation. All participants and their parents were informed of the purpose and procedures of the study. Participation in the study was voluntary and required the signing of written informed consent by participants and their parents.

To be eligible for recruitment, all participants (1) had to participate at least 8 h per week in off-road cycling, (2) had to participate in at least one competitive season, (3) had to perform an incremental test and power-cadence test at least once. All participants who suffered injuries in the six months prior to data collection were excluded.

The study, in accordance with the principles of the Declaration of Helsinki for the use of people in research, was approved by the Bioethics Committee of the University of Palermo (n. 217/2024 – prot. n. 66476-2024).

### 2.3 Procedures

All procedures were conducted during the competitive period by the same researcher in the Research and Development Technical Centre Laboratory of the Sicilian Regional Committee of the Italian Cycling Federation.

The participants, who were asked to avoid intense training in the 24 h prior to the test, were evaluated in two separate sessions 48 h apart. In the first session, a bike fitting was carried out with the aim of optimizing the participants’ joint functions and standardizing procedures. In the second session, the maturity offset of the participants was assessed through anthropometric measurements and, moreover, aerobic and anaerobic performance tests were administered.

#### 2.3.1 Bike fitting

Each participant was assessed on their own bike positioned on a specific roller (MagneticDays; Foiano della Chiana, Arezzo, Italy). To analyze the pedalling biomechanics, a 3D optical motion capture system (Cycling 3DMA, STT Systems; San Sebastián, Spain) was used. A saddle pressure analysis system (W-Saddle Pro, Sensor Products International S.R.L; Castel Maggiore, Bologna, Italy) was used to adjust the saddle optimally or to choose the most suitable saddle based on the characteristics of the participants (9, 10). At this point, each participant was asked to pedalling at an intensity of 2.8 Watts per kg (W*kg-1) of body weight at a cadence of 90 rpm for 2 min.

The 3D kinematic analysis and saddle pressures measurement suggested the necessary mechanical modifications to the bike. This procedure was repeated until joint function was optimized.

#### 2.3.2 Maturity offset

In the second session, 48 h later, the chronological age was calculated by subtracting each participant’s birth date from the test date as a decimal value to the nearest 0.1 year.

Standing height was measured using a standard stadiometer. Participants stood without shoes, with heels together, arms at their sides, and shoulders relaxed. Body weight was measured with participants standing without shoes and wearing only underwear (11). Sitting height was measured with participants seated on a box with their back against the wall, thighs fully supported on the bench, knees bent at 90°, feet on the floor, and hands resting on their thighs. To determine sitting height, the height of the box was subtracted from the vertical distance from the floor to the subject's head (2). Leg length was determined by subtracting sitting height from standing height (2).

Maturity status was calculated using the Mirwald's predictive equations, which estimates the maturity offset, that is, the number of years from the PHV (2). The Mirwald's predictive equations are as follows:

Maturity Offset for boys: −9.236 + (0.0002708 × Leg Length x Sitting Height) + (-0.001663 × Age x Leg Length) + (0.007216 × Age x Sitting Height) + [0.02292 × (Weight/Height)]

Maturity Offset for girls: −9.376 + (0.0001882 × Leg Length x Sitting Height) + (0.0022 × Age x Leg Length) + (0.005841 × Age x Sitting Height) + (−0.002658 × (Age x Weight) + (0.07693 × (Weight/Height)

Corresponding statistics were calculated relative to years before/after observed PHV: −3 = from −2.51 to −3.50; −2 = from −1.51 to −2.50; −1 = from −0.51 to −1.50; 0 = from −0.50 to 0.49; +1 = from 0.50 to 1.49; +2 = from 1.50 to 2.49; +3 = from 2.50 to 3.49 (3).

Each individual was also classified as pre-PHV, circa-PHV, or post-PHV maturity status using sex-specific mean observed ages at PHV ±1.0 year for the total samples of boys and girls, respectively.

#### 2.3.3 Aerobic and anaerobic performance tests

Each participant performed a warm-up for 10 min at a self-selected pedalling intensity and cadence.

All participants were asked to perform the power-cadence test, which consisted of five maximum sprints with different gear ratios (i.e., 50/12, 50/14, 50/16, 36/15, 36/17) in order to calculate the Peak Power Output (PPO). For each trial, all participants were asked to push all-out for 6 s to reach a maximal power. Among trials, all participants rested for 3 min.

Subsequently, an incremental ramp test was performed, starting with a power of 2.8 W*kg^−1^ of each participant's body weight with increments of 10 W*min^−1^ until exhaustion (12, 13). The Maximum Power Achieved (MPA) was the highest power maintained for one minute, which was the last step of the test before failure.

The relative and absolute PPO and MPA were considered for statistical analysis.

### 2.4 Statistical analysis

Data distribution was evaluated using the Shapiro–Wilk's test. Data were presented as means ± standard deviations.

A sensitivity power analysis for a multiple linear regression model with two predictors (alpha level of 0.05, sample size of 22 participants) was performed using G*Power (Version 3.1.9.7; Heinrich-Heine-Universität Düsseldorf, Düsseldorf, Germany).

A multiple linear regression analysis was conducted to determine whether maturity offset could be considered a predictor of aerobic and anaerobic performance outcomes, adjusting for sex. Specifically, the absolute PPO (W) and the relative PPO*kg^−1^, as well as the absolute MPA (W) and the relative MPA*kg^−1^, were examined as dependent variables. Maturity offset was set as the independent variable, and sex was included as covariate.

The Pearson’s correlation coefficient was conducted to measure the linear relationship between body weight and maturity offset.

Statistical significance was set at *p* < 0.05. All statistical analyses were performed using JAMOVI software package [The jamovi project (2024). jamovi (Version 2.5) [Computer Software]. Retrieved from https://www.jamovi.org].

## 3 Results

Participants’ characteristics are shown in (Table 1). According to the maturity status classification, 15 participants were classified as pre-PHV, 6 as circa-PHV, and 1 as post-PHV.

**Table 1:** Characteristics of the participants.

Parameter	Total	Male	Female
n	22	15	7
Age (years)	14.2 ± 1.0	14.5 ± 1.1	13.7 ± 0.8
Height (cm)	166.5 ± 10.5	170.0 ± 10.5	159.0 ± 2.4
Weight (kg)	54.9 ± 8.6	56.4 ± 9.6	51.7 ± 5.2
Maturity offset	−1.21 ± 1.08	−1.05 ± 1.24	−1.57 ± 0.37
MPA (W)	258.7 ± 55.8	281.0 ± 49.9	211.0 ± 23.4
MPA (W·kg⁻^1^)	4.71 ± 0.71	4.99 ± 0.54	4.11 ± 0.46
PPO (W)	795.2 ± 220.5	849.0 ± 205.0	680.0 ± 252.0
PPO (W·kg⁻^1^)	15.0 ± 2.5	15.0 ± 2.2	15.0 ± 3.3

Values are reported as mean ± SD. PPO, peak power output; MPA, maximal power achieved.

The sensitivity analysis showed that the study reached 80% power to detect large effect sizes (minimum detectable effect size: f^2^ = 0.52).

**Figure 1:**
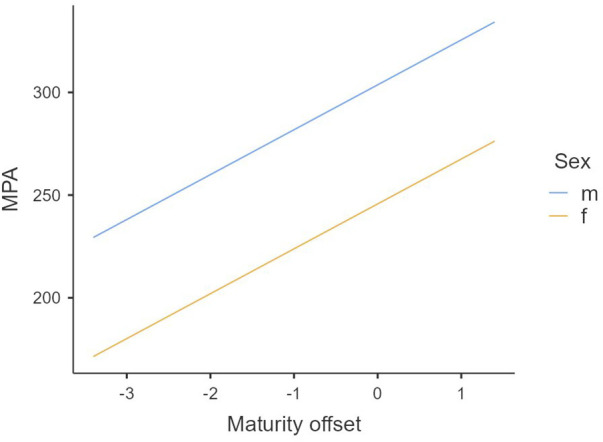
Multiple linear regression between maturity offset and absolute MPA, adjusted for sex. MPA, Maximum Power Achieved; m, males; f, females.

**Figure 2:**
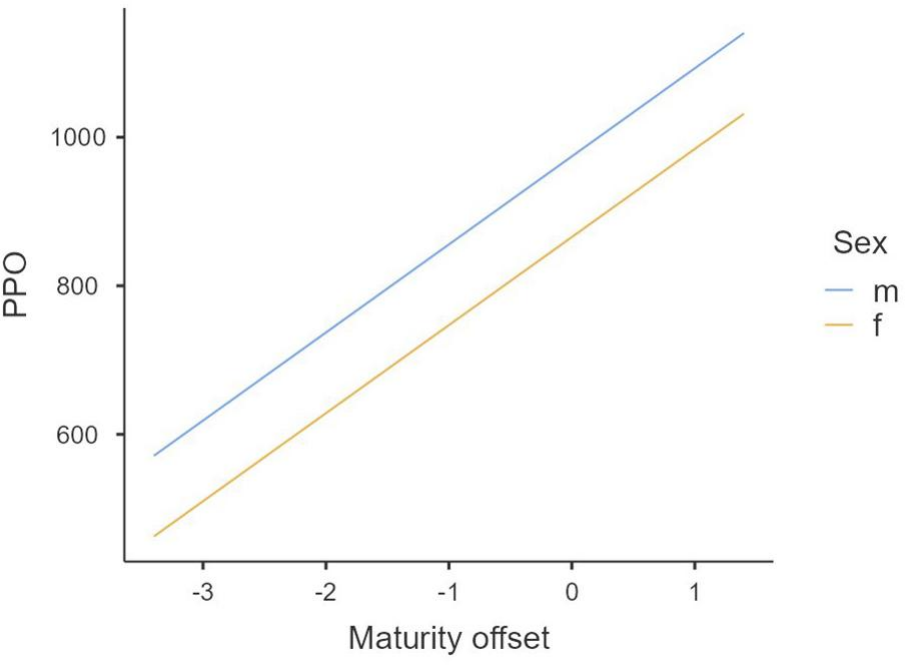
Multiple linear regression between maturity offset and absolute PPO, adjusted for sex. PPO, Peak Power Output; m, males; f, females.

The multiple linear regression analysis, adjusted for sex, showed that the maturity offset was a significant predictor of absolute PPO (R^2^ = 0.410, *p* = 0.007) and absolute MPA (R^2^ = 0.550, *p* = 0.013). Sex was significantly associated with MPA model (*p* = 0.004), while no association was observed for PPO model (*p* = 0.228) (Figures 1,2).

Our results showed that maturity offset was not a predictor of PPO*kg^−1^ (R^2^ = 0.058, *p* = 0.293) and MPA*kg^−1^ (R^2^ = 0.413, *p* = 0.665) (Figure 2). Sex was not associated with PPO·kg⁻^1^ (*p* = 0.792), but it was associated with MPA·kg⁻^1^ (*p* = 0.002).

The detailed regression results, including regression coefficients (B), standard errors (SE), standardized beta coefficients (*β*), 95% confidence intervals, and model fit indices, are presented in (Table 2).

**Table 2:** Multiple linear regression models adjusted for sex.

Outcome	Predictor	B	SE	*β*	95% CI	*p*-value	R^2^
PPO	Maturity offset	119	39.1	0.549	36.7; 200.4	0.007	0.410
PPO	Sex	−109.0	87.1	−0.473	−291.0; 73.8	0.228	0.410
PPO/kg	Maturity offset	0.580	0.536	0.248	−0.542; 1.700	0.293	0.058
PPO/kg	Sex	0.320	1.195	0.128	−2.182; 2.820	0.792	0.058
MPA	Maturity offset	21.9	8.02	0.431	5.06; 38.60	0.013	0.550
MPA	Sex	−58.0	17.89	−1.075	−95.40; −20.50	0.004	0.550
MPA/kg	Maturity offset	−0.049	0.112	−0.080	−0.283; 0.185	0.665	0.413
MPA/kg	Sex	−0.905	0.249	−1.377	−1.426; −0.384	0.002	0.413

B, regression coefficient; SE, standard error; β, standardized regression coefficient; CI, confidence interval; PPO, peak power output; MPA, maximal power achieved.

A significant positive correlation between body weight and maturity offset was found (r = 0.705, *p* <.001).

## 4 Discussion

The aim of this study was to analyze whether maturity offset could be a predictor of specific aerobic and anaerobic performance outcomes, such as PPO and MPA, in young off-road cyclists. The hypothesis was that a positive maturity offset could be a predictor of higher performance outcomes. Our results partially confirmed the hypothesis, because maturity offset represents a predictor of the absolute values of PPO and MPA, but not when these parameters were related to the participants’ body weight. Post-PHV participants showed maximum absolute power values in both the anaerobic and aerobic tests. However, it would appear that maturity offset was not associated with relative performance outcomes (W*kg^−1^). This result can be explained by the concomitant increase in body weight that occurs during biological maturation. Indeed, the significant positive correlation observed between body weight and maturity offset indicates that athletes post-PHV tend to have greater body weight. Therefore, although biological maturation is associated with improvements in absolute power production, these gains may be partially compensated when performance is normalized to body weight, resulting in no significant association between maturity offset and relative performance outcomes. Although maturity offset was estimated using sex-specific equations, sex independently contributed to some performance outcomes, particularly MPA and MPA·kg⁻^1^. This indicates that maturity status and sex have distinct effects on performance during adolescence and should be considered separately.

These data are consistent with those reported by several authors who have highlighted a marked influence of maturity status on physical performance (14). In particular, anaerobic performances, such as sprinting, jumping, and short-term power output, have frequently been associated with an advanced degree of biological maturation, thanks to increased muscle mass and fibre cross-sectional area (1, 14–19). Our results are consistent with the study by Mendez-Villanueva et al. (2011) who analyzed the possible influence of biological maturation and anthropometry on three speed-related qualities in different age groups of young male soccer players (16). Their results suggested that the positive effects of age on running speed qualities during growth were more related to biological maturation processes rather than to anthropometric characteristics (16). Similarly, in a recent study by Scardina et al. (2026) in a sample of young soccer players, authors demonstrated that post-PHV players had significantly higher performance in several outcomes including jumping, sprinting, aerobic capacity, and lower limb peak power (20). The study by Meyers et al. (2015), which aimed to examine the natural development of the mechanical determinants of maximal sprint speed in relation to maturation in a large sample of boys, indicated that the period around PHV represents a key phase for improvements in sprinting performance (17). Similar to our findings, Rumpf et al. (2013) investigated whether vertical and leg stiffness, along with stretch-shortening cycle capacity, are variables that play a key role in sprint performance, vary among developing athletes at different stages of maturity during maximal sprinting (18). The authors found that maturity affects the ability to absorb and produce power and that these mechanical factors are predictors of maximum running speed (18). Additionally, Philippaerts et al. (2006) investigated the correlation between PHV and physical performance in youth football players (1). Their study demonstrated that balance, speed of limb movement, trunk strength, upper-body muscular endurance, explosive power, running speed and agility, cardiorespiratory fitness, and anaerobic capacity reach their peak during the period of PHV (1).

Our results indicate that maturity offset is a predictor of absolute performance, but it does not predict relative measures like the power-to-weight ratio, which is crucial in cycling. For example, Meyers et al. (2017) found that in post-PHV boys, mass negatively influenced maximal sprint performance (21). Similarly, Moran et al. (2017) observed a strong correlation between maturity offset and both strength and sprinting in absolute terms, although these relationships are weaker when considering that relative values could be insignificant because, as young people grow, relative strength may decrease as height and body weight increase (22). In contrast to our findings, the study by Armstrong et al. (2001) aimed to investigate changes in Mean Power Output and PPO related to age, body size, fatness, sex, and maturity in boys and girls with an average age of 12.2 years (19). Their results confirmed that body mass and skinfold thicknesses are significant on short-term power output, but maturity does not have a significant effect on either peak or mean power as measured by the Wingate anaerobic test (19).

Regarding aerobic performance, previous studies have shown that maturation and absolute aerobic power are significantly related (23, 24). In our sample, the maturity offset also correlated with aerobic outcomes, suggesting that maturity play a role in improving aerobic performance. In line with our findings, Gundersen et al. (2026) conducted a recent study on how growth and biological maturation affect physical performance, such as aerobic capacity, in youth soccer players aged 14.1 to 17.4 (25). Their results showed that biological maturation had a significant link with VO_2_max during early adolescence, but this connection decreased as the players aged. These findings suggest that the influence of maturation on aerobic performance is especially significant around PHV (25). Our findings align with those by Carvalho et al. (2013), who examined how chronological age, biological maturation, training experience, and body size were related to VO_2_max in male basketball players aged 14–16 (26). Their results indicated that biological maturity and training experience significantly impacted VO_2_max (26). In agreement with our findings, Fernández-Jávega et al. (2025) investigated the influence of biological maturation on training load and performance adaptations following a running-based high-intensity interval training program in youth football players (27). The study revealed that post-PHV players exhibited higher baseline intermittent endurance performance compared with pre-PHV athletes (27). The study by Almeida-Neto et al. (2022) investigated the influence of biological maturation on aerobic and anaerobic performance in young rowing athletes (8). While biological maturation did not significantly affect relative VO_2_max, it was linked to higher absolute aerobic power and improved anaerobic performance (8). This suggests that maturity status influences the development of energy systems and overall endurance capacity. The results of the study by Gundersen et al. (2022), which investigated the biological maturation level and physical capacity in male youth soccer players, are in line with ours, showing that biological maturation does not significantly influence absolute VO_2_max after adjusting for subjects’ body weight (28).

## 5 Conclusions

Maturity status is an important contextual factor in interpreting aerobic and anaerobic performance in young off-road cyclists during adolescence and can help professionals avoid distortions related to maturity, but it should not be interpreted as a direct indicator of future athletic talent or potential.

### 5.1 Practical implications

Sports scientists, coaches, and practitioners should use caution when comparing young athletes of similar chronological age, as differences in maturity status can influence absolute performance outcomes. These findings highlight the importance of assessing maturity status when evaluating the physical performance in young athletes, as well as for monitoring the biological developmental trajectories of young athletes.

### 5.2 Strengths and limitations

One of the main strengths of this study is the use of sport-specific testing protocols performed on the athletes’ own bicycles combined with bike-fitting procedures, which, by optimizing joint function for each participant, reduce variability in power output assessment. Evaluating both aerobic and anaerobic performance through a power-cadence test and an incremental ramp test provided a comprehensive overview of young off-road cyclists’ performance capabilities. Furthermore, focusing on young off-road cyclists represents an additional strength, as this population has been relatively less studied in the literature. Finally, the distinction between absolute and relative performance outcomes allowed for a more discipline-specific interpretation of the influence of maturity status on cycling performance.

However, some limitations must be acknowledged. The small sample size, particularly the limited number of female participants, may limit the generalizability of the findings and prevent robust sex-specific analyses. Nevertheless, sex was included as a covariate in the regression models to partially account for potential sex-related differences. Moreover, maturity status was calculated using the Mirwald's equation, which is an estimation of the maturity offset. This method provides an estimate of maturity timing and is subject to systematic prediction error, particularly near PHV, which should be considered when interpreting associations. Therefore, these results should be interpreted with caution, and given the relatively small sample size of this study, even minor inaccuracies in the estimation of maturity may have influenced the strength of the observed associations. Furthermore, the cross-sectional study design precludes any causal interpretation of the observed associations. Longitudinal studies are needed to better understand how biological maturation influences performance development over time. Another limitation is the lack of body-composition assessment. Since body weight appears to play an important role in the relationship between biological maturation and performance outcomes, the inclusion of lean and fat mass measurements could provide additional insight into the physiological mechanisms underlying these associations.

## Data Availability

The raw data supporting the conclusions of this article will be made available by the authors, without undue reservation.
